# Immune Efficacy of a Genetically Engineered Vaccine against Lymphocystis Disease Virus: Analysis of Different Immunization Strategies

**DOI:** 10.1155/2011/729216

**Published:** 2011-07-19

**Authors:** Fengrong Zheng, Xiuqin Sun, Xing'an Wu, Hongzhan Liu, Jiye Li, Suqi Wu, Jinxing Zhang

**Affiliations:** ^1^First Institute of Oceanography, State Oceanography Administration of China, 6 Xianxialing Road, Qingdao City, Shandong Province 266061, China; ^2^Department of Microbiology, Fourth Military Medical University, 17 Changlexi Road, Xi'an City, Shanxi Province 710032, China; ^3^Marine College, Shandong University, Weihai 264209, China

## Abstract

Here, we report the construction of a vaccine against lymphocystis disease virus (LCDV) using nucleic acid vaccination technology. A fragment of the major capsid protein encoding gene from an LCDV isolated from China (LCDV-cn) was cloned into an eukaryotic expression vector pEGFP-N2, yielding a recombinant plasmid pEGFP-N2-LCDV-cn0.6 kb. This plasmid was immediately expressed after liposomal transfer into the Japanese flounder embryo cell line. The recombinant plasmid was inoculated into Japanese flounder via two routes (intramuscular injection and hypodermic injection) at three doses (0.1, 5, and 15 **μ**g), and then T-lymphopoiesis in different tissues and antibodies raised against LCDV were evaluated. The results indicated that this recombinant plasmid induced unique humoral or cell-mediated immune responses depending on the inoculation route and conferred immune protection. Furthermore, the humoral immune responses and protective effects were significantly increased at higher vaccine doses via the two injection routes. Plasmid pEGFP-N2-LCDV0.6 kb is therefore a promising vaccine candidate against LCDV in Japanese flounder.

## 1. Introduction

Nucleic acid immunization, based on the introduction of plasmid DNA encoding a protective antigen into animal tissue, can express the plasmid-encoded protein and induce subsequent immune responses [1]. Much effort has been invested in this technology since gene engineering vaccines possess multiple advantages over killed, attenuated, or subunit vaccines [2]. Indeed, gene engineering vaccines are known to stimulate both nonspecific and specific immune responses without the need for live organisms, replicating vectors or adjuvants [3]. Antigen synthesis induced by nucleic acid vaccination imitates natural infection by intracellular pathogens and leads to subsequent cell-mediated responses and ultimately, the generation of memory lymphocyte responses [4]. Additionally, gene engineering vaccines have already been shown to provide protection for fish to various intracellular pathogens, such as viral hemorrhagic septicemia and infectious hematopoietic necrosis virus [5, 6]. Anderson et al. (1996) reported the first application of gene engineering vaccine technology where a plasmid containing the glycoprotein (G) gene of IHNV was used to stimulate a protective immune response in rainbow trout fry [7]. Furthermore, several studies have shown that a nucleic acid vaccine against IHNV provides significant protection in rainbow trout against either waterborne or injection challenges in fish that range in size from 2 to 160 g [8–10]. Traxler et al. (1999) have reported significantly high levels of protection against IHNV also observed in vaccine efficacy studies in Atlantic salmon, other economically important species [11]. A Nucleic acid vaccine containing the G gene of other rhabdoviral pathogen of rainbow trout, viral hemorrhagic septicemia virus (VHSV), has also been shown to provide significant protection when administered alone or in combination with a nucleic acid vaccine against IHNV [5, 6, 12].

Studies regarding nucleic acid vaccines for fish published in recent years have mainly focused on infectious hematopoietic necrosis virus (IHNV) [8, 9, 12–16], viral hemorrhagic septicemia virus (VHSV) [5, 6, 12, 17–19], hirame rhabdovirus (HIRRV) [20], herpesvirus (IHV-1) [21], infectious pancreatic necrosis [22], red sea bream iridovirus (RSIV), and spring viraemia of carp virus [23]. However, research regarding lymphocystis disease virus (LCDV), the causative agent of lymphocystis disease (LCD), a common chronic disease among many salt and fresh water fish species, remains limited. LCD occurs worldwide, and the rate of incidence appears to be increasing [24], severely affecting the fish farming industry. 

We previously constructed two genetically engineered vaccines against LCDV for the prevention and control of LCD [25, 26] and investigated the distribution and expression of immune-related genes in Japanese flounder (*Paralichthys olivaceus*) after immunization with the vaccines [26, 27]. In this study, we investigated the optimal inoculation routes and doses for these vaccines in Japanese flounder.

## 2. Materials and Methods

The FG-9307 cell line from Japanese flounder gills and the flounder embryo cell (FEC) line from Japanese flounder were obtained from Dr. Shangliang Tong, Ocean University of China and Dr. Songlin Chen, Yellow Sea Fisheries Research Institute Chinese Academy of Fishery Sciences, respectively. The two cell lines were maintained in minimum essential medium (MEM) and Dulbecco's modified Eagle's medium (DMEM), respectively. Culture medium was supplemented with 15% heat-inactivated fetal bovine serum (FBS), 2 mM L-glutamine, 50 IU/mL penicillin, 50 mg/mL streptomycin (Cellgro, USA), and 1% nonessential amino acids (Cellgro, USA), buffered to pH 7.4 with 7.5% sodium bicarbonate. The fish FG-9307 and FEC lines were maintained at 22°C and 24°C, respectively.

Tumors obtained from infected fish were wiped to remove the connective tissue and then freeze-thawed three times and centrifuged at 3000 g for 15 min. The cell suspension was then loaded onto a 20–60% sucrose gradient and centrifuged at 20,000 g for 2 h. The virus was observed using a photomicroscope, and the virus concentration was determined using a spectrophotometer.

LCDV was propagated in the FG-9307 cell line [28, 29]. The culture medium was harvested when viral cytopathic effects were apparent, and the clarified crude virus was stored at −80°C until use.

### 2.1. Clone, Identification, and Sequence Analysis of 0.6 kb Fragment

Viral DNA was extracted from LCDV samples following the manufacturer's instructions (OMEGA, USA). DNA was precipitated with 100% ethanol, washed three times with 70% ethanol, air dried, and suspended in 40 *μ*L sterile, distilled, and autoclaved water. DNA concentration was estimated using a spectrophotometer.

A fifty-microliter PCR reaction mixture consisting of 5 *μ*L DNA, 0.05 nmol of each primer, 4 *μ*L 2 mM MgCl_2_, 2.5 units* Taq* DNA polymerase, 4 *μ*L 2 nM dNTP, 5 *μ*L PCR buffer, and ultrapure water 26 *μ*L was prepared. A primer pair flanking the *Mcp* gene, the forward (5′-GAC GAA TTC ATG ATC GGT ATT AC-3′), and the reverse (5′-GAC GCG GCC GCG AAT AAT ATT CAC T-3′) primers were used. Amplification was performed at 94°C for 4 min, followed by 28 cycles of 1 min denaturation at 94°C, 45 s of annealing at 50°C, and 45 s of extension at 72°C, and a final extension at 72°C for 10 min.

### 2.2. Construction of Gene Engineering Vaccine against Lymphocystis Disease Virus

The gene encoding ORF 0147L of the major capsid protein (MCP), approximately 0.6 kb in length, and the eukaryotic expression vector pEGFP-N2 (Invitrogen) were verified by *Eco*RI and *Sal *I, respectively. The 0.6 bp fragment was cloned into the expression vector pEGFP-N2, behind the cytomegalovirus promoter and yielded EGFP-N2-LCDV0.6 kb.

### 2.3. Transfection of the Eukaryotic Expression Vector and Evaluation of Expression

Cell transfection was performed using Lipofectamine, in eukaryotic FEC, following the manufacturer's instructions (Gibco BRL). The cells were maintained at 24°C for 48 h after transfection. Fluorescent microscopy and RT-PCR were employed to evaluate the immediate expression of pEGFP-N2-LCDV-cn0.6 kb in the FEC line.

### 2.4. Preparation of Plasmid DNA

The recombinant plasmid pEGFP-N2-LCDV-cn0.6 kb was verified by digestion with restriction endonucleases *Xho*I and *Bam*HI and then transformed into *E. coli *DH5*α*. The recombinant plasmid (DNA vaccine) was prepared on a large-scale, distilled, and purified by resin using the Endo Free Plasmid Kit (Promega) according to the manufacturer's instructions. The DNA was then suspended in PBS and stored at −20°C. The quality and quantity of the DNA were determined by spectrophotometry.

### 2.5. Vaccination of Fish

LCDV free Japanese flounder fish, approximately 15–20 cm in body length and approximately 60–80 g in body weight, were used as fish to evaluate the vaccine function of plasmid DNA. The fish were obtained from a cultivation farm and kept in a tank with a flowthrough, filtered and virus free water system at approximately 18–22°C with water quality monitored daily. They were fed with commercially available dry feed pellets corresponding to 3–5% of total body weight, twice per day. Prior to vaccination, the fish were acclimatized for 2 weeks in the laboratory.

Fish (*n* = 600 per group) were randomly selected and anaesthetized using 0.02% tricaine methanesulfonate (MS-222). Fish were injected to a depth of 8 mm into the left epaxial muscle immediately anterior to the dorsal fin, using an insulin syringe and a 29 G needle. The experimental fish were divided into 11 groups: (1) control fish, (2) 100 *μ*L phosphate-buffered saline (pH 7.4; PBS) via intramuscular injection (i.m.), (3) 5 *μ*g pEGFP-N2 via i.m., (4) 0.1 *μ*g pEGFP-N2-LCDV-cn0.6 kb via i.m., (5) 5 *μ*g pEGFP-N2-LCDV-cn0.6 kb via i.m., (6) 15 *μ*g pEGFP-N2-LCDV-cn0.6 kb via i.m., (7) 100 *μ*L PBS via hypodermic injection (i.h.), (8) 5 *μ*g pEGFP-N2 via i.h., (9) 0.1 *μ*g pEGFP-N2-LCDV-cn0.6 kb via i.h., (10) 5 *μ*g pEGFP-N2-LCDV-cn0.6 kb via i.h., and (11) 15 *μ*g pEGFP-N2-LCDV-cn0.6 kb via i.h. Plasmid DNA was dissolved in 100 *μ*L of PBS. After vaccination, each group of 60 fish was kept in different tanks under the same experimental conditions.

### 2.6. Lymphoproliferative Assay

#### 2.6.1. Preparation of Blood Lymphocytes from Fish

A 0.5 mL blood sample was isolated from the caudal sinus of fish in a sterile coequal anticoagulant in 2.5 mL of lymphoprep separation medium (Solarbio, Beijing, China). The coat layer was collected, washed twice in cold RPMI-1640 medium, and resuspended. The cells were then adjusted to 1 × 10^6^ cells/mL with RPMI-1640 medium supplemented with 2 mM L-glutamine, 10% heat-inactivated FBS, 50 IU/mL penicillin, 50 mg/mL streptomycin, and 1% nonessential amino acids.

#### 2.6.2. The Preparation of Anterior Kidney, Spleen, and Hind Intestines Lymphocytes from Fish

Anterior kidney, spleen, and hind intestines lymphocytes from fish in each control and vaccination group were collected aseptically by removing all tissues and placing the organ in a Petri dish containing 10 mL sterile RPMI-1640 medium (Hyclone, USA). Cells were released from all tissues by mechanical disruption using a curved forceps and a metallic sieve screen (200 *μ*m). The resulting cell suspension was washed twice in RPMI-1640 medium and resuspended in 3 mL RPMI-1640 medium and centrifuged at 1500 g for 15 min. The coat layer was collected, washed twice in cold RPMI-1640 medium, and resuspended. The cells were then adjusted to 1 × 10^6^ cells/mL with RPMI-1640 medium supplemented with 2 mM L-glutamine, 10% heat-inactivated FBS, 50 IU/mL penicillin and 50 mg/mL streptomycin (Cellgro), and 1% nonessential amino acids (Cellgro).

**Lymphoproliferative Assay**

Cells (500 *μ*L) were cultured in triplicate in 24-well plates (Corning, NY) at 22°C in 5% CO_2_ with 2 *μ*L LCDV-cn (19.8 mg/mL), or no additives (negative control). The cells were cultured at 22°C in 5% CO_2_ for 48 h. After 48 h, 200 *μ*L Thiazolyl blue (Genview) was added to each well. The cells were incubated for a further 4 h, DMSO was then added to the wells at 500 *μ*L/well, and the absorbance was measured at 570 and 600 nm using a kinetic microplate reader (Molecular Devices). The Thiazolyl blue assay was developed as a nonradioactive lymphocyte proliferation assay, which indirectly measures cell proliferation. The level of proliferation is indicated by the difference between the specific absorbance of the oxidized form (570 nm) and the reduced form (600 nm). The specific absorbance of the unstimulated cells (negative control) is subtracted from the specific absorbance of the cells to yield a delta-specific absorbance.

### 2.7. Determination of Serum Antibody Levels

Japanese flounder blood samples (1 mL) were collected on days 21, 35, 56, and 90 p.i. with a syringe from the caudal sinus of nine of the eleven groups and allowed to clot at 20°C for 20 min, then 4°C for 12 h. Serum was obtained after centrifugation at 500 g to remove cell particulate matter and stored at 80°C for further study.

The antibody responses of the fish from each group were evaluated for the presence of specific immunoglobulin against LCDV using an indirect ELISA. LCDV was diluted to a 100 *μ*g/mL concentration in bicarbonate coating buffer (pH 9.6) and the solution was used to coat polystyrene plates with 100 *μ*L/well. The plates were incubated at 4°C overnight, washed four times with wash buffer (Tris-buffered saline (TBS) at pH 7.4, 0.05% Tween 20), and blocked with 2% BSA in TBS for 2 h at room temperature. The blocking solution was then removed, and diluted fish serum samples (1 : 80 dilution in blocking solution) were added to individual triplicate wells at 100 *μ*L/well. A positive control serum sample and a diluent only sample were tested in the same manner. The plates were incubated for 90 min at 37°C and then washed four times with wash buffer. The secondary antibody solution, a protein peroxidase conjugate (Sigma), was added at 100 *μ*L/well at a 1 : 1500 dilution. After 90 min at 37°C, the plates were washed four times, and 100 *μ*L of substrate solution (TMB Microwell peroxidase substrate; Kirkegaard & Perry Laboratories, Gaithersburg, MD) was added to each well. After 20 min of incubation at room temperature, 100 *μ*L of stop solution (2 mol/L sulfuric acid) was added. The absorbance at 450 nm was then recorded using a microplate reader (Microplate reader Benchmark, Bio-Rad Laboratories, s.r.1. Milano, Italy). Each serum sample was compared with the control wells.

**Challenge Experiment**

The experimental fish were divided into four groups: (1) 100 *μ*L PBS, (2) 5 *μ*g pEGFP-N2, (3) 5 *μ*g pEGFP-N2-LCDV-cn0.6 kb via i.m., and (4) 5 *μ*g pEGFP-N2-LCDV-cn0.6 kb via i.h. After vaccination, each group was kept in a different tank under identical experimental conditions. Twenty-one days after vaccination, fish were placed in tanks and infected with LCDV. The fish were then observed, and the growth of tumors was noted after one and two months.

### 2.8. Statistical Analysis

Results from ELISA and lymphoproliferative assay data were subjected to a mixed model repeated analysis of variance, and SPSS software was employed to compare the various experimental groups each day. The data for each test was reported as the mean ± S.E.M. An overall level of significance with *P* < 0.05 was accepted.

## 3. Results

### 3.1. Construction and Identification of the Eukaryotic Expression Vector

The DNA vaccine pEGFP-N2-LCDV-cn0.6 kb was verified by *Xho*I and *Bam*HI endonuclease restriction analysis to contain the desired DNA fragment and associating elements. The plasmid was prepared, purified, and suspended in endotoxin-free water. The 0.6 kb MCP sequence is shown in Table 1.

### 3.2. The Detection of Immediate Expression of the Plasmid in the FEC Line by Fluorescent Microscopy

**Figure 1:**
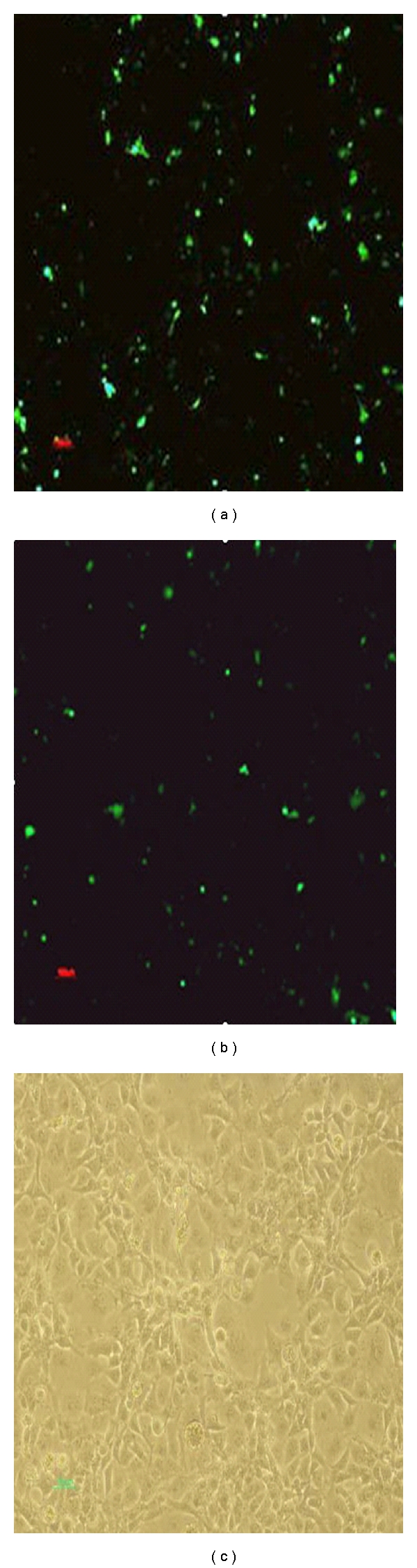
Fluorescent and optical microscopy images of cells transfected with pEGFP-N2-LCDV-cn0.6 kb and pEGFP-N2 plasmid DNA. (a) Fluorescent microscopy image of pEGFP-N2-LCDV-cn0.6 kb; (b) fluorescent microscopy image of pEGFP-N2; (c) optical microscopy image of pEGFP-N2-LCDV-cn0.6 kb.

**Figure 2:**
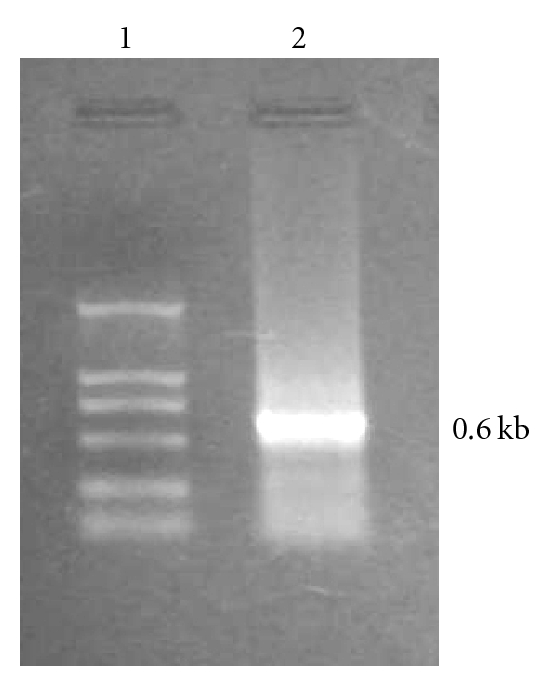
The detection of flounder embryo cells (FECs) transfected by pEGFP-N2-LCDV-cn0.6 kb by RT-PCR. (1) DL2000 DNA marker; (2) 0.6 kb fragment.

Fluorescent microscopic images of the expression of the FEC cell-transfected plasmid DNA, pEGFP-N2-LCDV-cn0.6 kb, are shown in Figure 1. The image clearly shows that the transfected cells emitted fluorescence, whereas the control untransfected cells did not. The RT-PCR results are shown in Figure 2.

### 3.3. Lymphoproliferative Detection Assay

**Figure 3:**
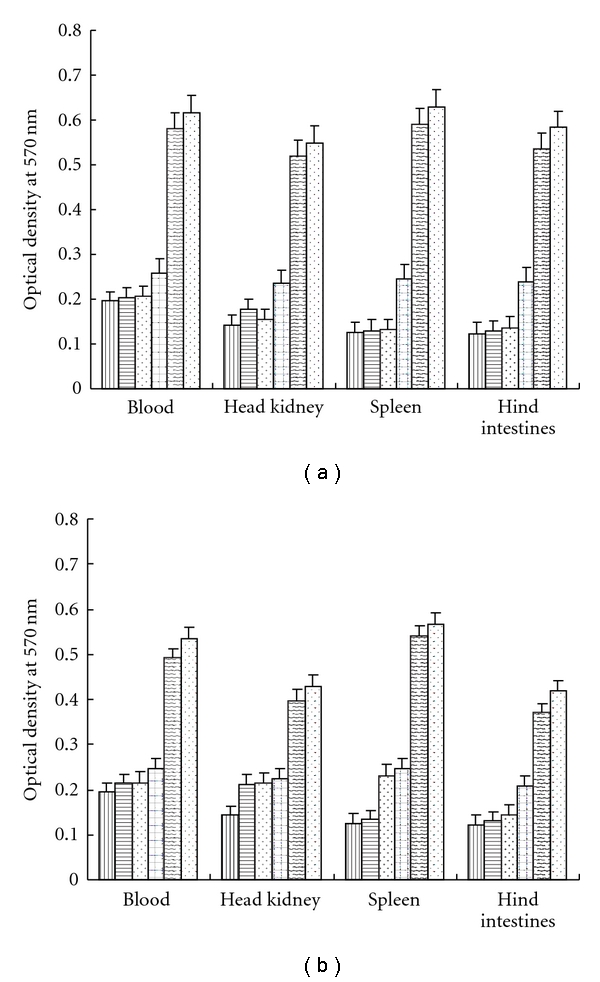
Proliferation of tissue lymphocytes from all groups after *in vitro *stimulation with LCDV. (a) Intramuscular injection; (b) hypodermic injection. Cells were harvested on day 21 and cultured for two days. Control group (vertical bar); PBS group (horizontal bar); 5 *μ*g pEGFP-N2 group (triangular bracket); 0.1 *μ*g pEGFP-N2-LCDV-cn0.6 kb group (pane); 5 *μ*g pEGFP-N2-LCDV-cn0.6 kb group (wave bar); 15 *μ*g pEGFP-N2-LCDV-cn0.6 kb group (dot). Results are shown as the mean ± S.E.M. of the OD_450_ values. Significant differences (*P* < 0.05) were observed between the pEGFP-N2-LCDV-cn0.6 kb group and the no-injection groups, and the PBS and pEGFP-N2 groups.

Lymphocytes of tissues from all of the groups were cultured *in vitro*, following LCDV stimulation, and significant lymphoproliferative responses were detected on day 21 after vaccination in the peripheral blood, spleen, head, kidney, and hind intestine of all vaccination groups. The level of the response increased with the dose, but no significant difference was observed between the 5 *μ*g and 15 *μ*g doses. Lymphoproliferative responses were found to be particularly high in the peripheral blood and hind intestine samples (Figure 3). No antigen-specific lymphoproliferative responses were detected in the pEGFP-N2 or saline groups. These results indicated that plasmid pEGFP-N2-LCDV-cn-MCP0.6 kb has the ability to enhance specific cellular responses, with significantly greater lymphocyte responses detected among the i.m. groups compared with the i.h. groups.

### 3.4. Antibody Production in the Vaccinated Fish

**Figure 4:**
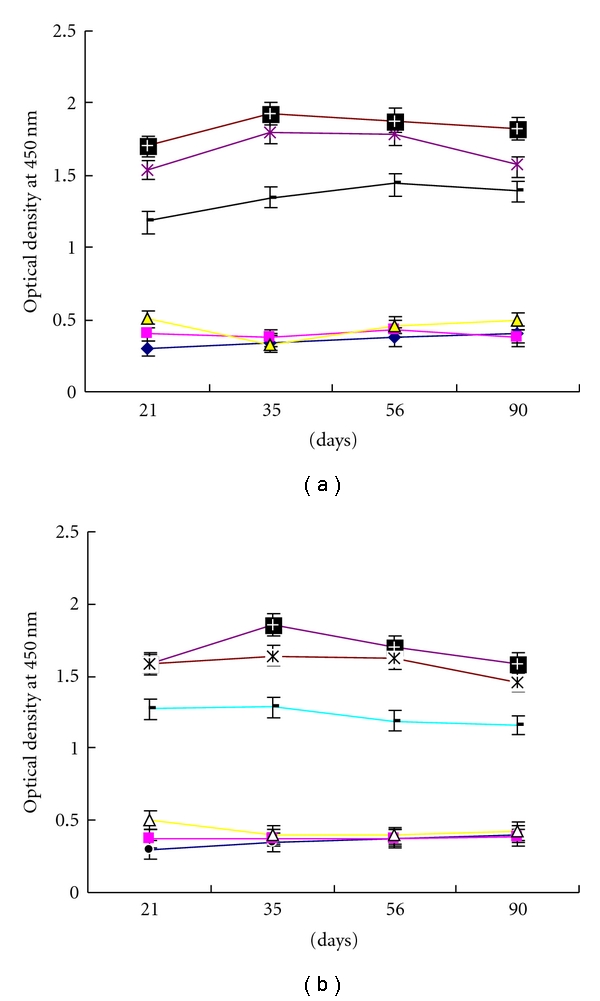
Detection of LCDV-specific antibodies from the sera of DNA-vaccinated Japanese flounder collected on days 21, 35, 56, and 90 after vaccination by ELISA. (a) Intramuscular injection; (b) hypodermic injection. 15 *μ*g pEGFP-N2-LCDV-cn0.6 kb group (plus sign); 5 *μ*g pEGFP-N2-LCDV-cn0.6 kb group (asterisk); 0.1 *μ*g pEGFP-N2-LCDV-cn0.6 kb group (horizontal line); pEGFP-N2 group (triangle); PBS group (square); no injection (block dot). Results are shown as the mean ± S.E.M. of the OD_450_ values.

The antibody response of each group was evaluated for the presence of specific immunoglobulin against LCDV using an indirect ELISA (Figure 4). Low levels of LCDV-specific antibodies were detected in all of the pEGFP-N2-LCDV-cn0.6 kb-vaccinated fish after three weeks, and antibody levels increased along with the dose. Increasing concentrations of antibodies were generated up to 35 days after vaccination, with the greatest increase observed following a booster vaccination on day 21. Significantly greater responses were observed in the 5 and 15 *μ*g groups than in the 0.1 *μ*g group, and there were no significant differences between these former two groups. After day 56, the concentration of antibodies began to decline, though the fish maintained relatively high levels of antibodies until day 90. Slightly higher responses were seen among the i.h. groups than the i.m. groups on day 21, but the antibody levels in the i.h. groups were lower than in the i.m. groups after 35 days, and this phenomenon persisted after 90 days.

### 3.5. Protection against LCDV

The protection yielded by recombinant plasmid pEGFP-N2-LCDV-cn0.6 kb is shown in Table 2. One month after challenge, the efficiency of tumor growth in the PBS group, the pEGFP-N2 group, and the pEGFP-N2-LCDV-cn0.6 kb-vaccinated groups was 22.4%, 19.6%, 2.6%, and 2.4%, respectively. The tumors were small and mainly grew in the mouth. Two months after challenge, the efficiency of tumor growth in the groups listed above was 32.6%, 32.1%, 3.17%, and 3.21%, respectively, and the tumors were large and existed throughout the whole body, spreading from the mouth and gills to the fins.

## 4. Discussion

The development of genetically engineered vaccines for fish has been increasingly studied in recent years, and such vaccines have been shown to provide protection in fish against various intracellular pathogens, such as VHSV and IHNV [5, 6]. The fact that these vaccines successfully induced a protective immune response against intracellular pathogens suggested that a genetically engineered vaccine against LCDV infection was also feasible; however, until now, this possibility had not been widely studied. In the present study, we analyzed the MCP gene (01470.6-kb) of LCDV-cn, which encodes 71696–72318 amino acids, and revealed a 0.6 kb antigenic fragment. This fragment was cloned into the prokaryotic expression vector pCI-neo and was found to elicit specific responses to polyclonal antiserum against LCDV. The eukaryotic expression vector pEGFP-N2, containing the GFP gene, was used in our experiments under the control of the CMV promoter. We demonstrated that a genetically engineered vaccine encoding the LCDV MCP gene elicited significant levels of protective LCDV-specific immunity, the levels of which were dose dependent and roughly proportional to the amount of protection conferred.

We analyzed vaccination strategies based on two injection routes, intramuscular injection and hypodermic injection, and three injection doses, 0.1, 5, and 15 *μ*g of naked circular plasmid DNA. These selected doses fall within the range of plasmid DNA (1–50 *μ*g) routinely used to express foreign genes in fish muscle and were found to be adequate to induce antigen-specific immune responses in 60–80 g Japanese flounder. At this preliminary stage, no attempt was made to evaluate the effects of intramuscular injection using different doses of DNA, which has already been detailed for other fish species [1, 28–32]. Specific experiments based on Japanese flounder biology are required to address each of these points prior to potentially applying these vaccines to farmed fish. 

In a previous study in goldfish, antibodies against *β*-galactosidase were detected as early as seven days after injection of LacZ-encoding DNA, and the antibody response lasted for at least 10 weeks although the number of antibody-producing cells appeared to decline rapidly [30]. In rainbow trout, antibodies to the VHSV G protein were detected 23 days after injection with a plasmid encoding the G gene, and serum antibodies to the G protein of IHNV were detected 3 to 15 weeks after inoculation [5, 7]. The results of the present study showed that injection of naked plasmid DNA containing the MCP gene induced an efficient, systemic, and antigen-specific immune response in Japanese flounder, with detectable anti-LCDV antibody levels in fish 21 days after injection. 

Some differences were found in vaccine efficiency when comparing the three vaccine doses. Low levels of specific antibodies to LCDV were detected in all pEGFP-N2-LCDV-cn0.6 kb-vaccinated fish three weeks after inoculation, and the antibody level increased with the increasing dose. Significant protective immune responses were generated following administration of the 15 and 5 *μ*g doses, but not the 0.1 *μ*g dose on day 21, indicating that the 5 *μ*g dose was more efficient than the 15 *μ*g dose when considering overall protection. No specific antibody responses were detected in the PBS or pEGFP-N2 groups. 

Although the specific immune responses varied according to dose, a different effect was exhibited when the nonspecific respiratory burst was evaluated. However, in the present study, the induction of a respiratory burst increased after vaccination, but no difference was observed between the control and vaccinated groups.

In conclusion, our results strongly suggested that both humoral and cellular responses were stimulated by the vaccine. These initial findings indicate the potential for the development of a protective vaccine against LCDV.

## Figures and Tables

**Table 1:** The 0.6 kb MCP sequence of ORF0147 (71318–71696 amino acids).

ATGATCGGTAATACTATTGATATGACACAACCCGTTGATTCCAATGGTCAATT
ACCTGAAGAAGTGTTAATACTTCCTTTACCTTATTTCTTTTCTCGAGATAGCG
GTATGGCTTTACCCAGCGCTGCTTTGCCTTATAATGAAATAAGATTAACTTTT
CATCTGAGAGATTGGACTGAATTATTGATCTTTCAAAATAAAAACGACTCTA
CCATCATGCCTTTGACAGCAGGCGATTTAGACTGGGGTAAACCTGATTTAA
AGGATGTGCAAGTATGGATTACTAATGTAGTAGTAACCAATGAGGAACGTC
GTTTAATGGGTACAGTACCTAGAGACATCTTGGTGGAACAGGTACAAACAG
CACCTAAACATGTATTTCAACCTCTAACTATTCCAAGTCCTAATTTTGACATC
AGATTTTCTCATGCCATTAAAATCCTTTTTTTCGGTGTGCGTAATGTTACCTA
TCAAGCTATACAATCCAATTACACCAGTTCTTCTCCTGTAATCTTTGACGGT
GGAATTGCTAGCGATTTACCGGGTATTGCTGCTGATCCTATTTCAAATGTTAC
CTTGGTTTATGAAAATAGTGCTCGTCTTAATGAAATGGGTAGTGAATAT

**Table 2:** The efficiency of tumor growth in the different groups of fish, one and two months after injection.

	PBS group	pEGFP-N2 group	Intramuscular injection 5 *μ*g/fish group	Hypodermic injection 5 *μ*g/fish group
The amount with tumour 1 month (fish)	112	98	26	24
The total amount 1 month (fish)	500	500	1000	1000
The efficiency of tumour growth	22.4%	19.6%	2.6%	2.4%
The amount with tumour 2 months (fish)	158	152	31	31
The total amount 2 months (fish)	484	473	978	967
The efficiency of tumour growth	32.6%	32.1%	3.17%	3.21%
